# Jejunal Angiodysplasia as an Unusual Cause of Hemorrhage After Hepaticojejunostomy

**DOI:** 10.7759/cureus.62502

**Published:** 2024-06-16

**Authors:** Shiva S, Pankaj Kumar, Amit Karnik, Awanish Kumar, Abhinav A Sonkar

**Affiliations:** 1 Surgery, King George’s Medical University, Lucknow, IND

**Keywords:** post-hepaticojejunostomy hemorrhage, angioembolization, postoperative hemorrhage, ectopic varices, jejunal angiodysplasia

## Abstract

Ectopic variceal bleeding is a rare cause of postoperative hemorrhage following hepaticojejunostomy and should be differentiated from other causes such as pseudoaneurysms or ulcers. Uncommon complications post-hepaticojejunostomy demand scrupulous attention, and this case report reveals a seldom-documented scenario of jejunal angiodysplasia as an elusive cause of postoperative bleeding. Through a comprehensive examination of the patient’s clinical trajectory, diagnostic challenges, and subsequent management, this report contributes to the expanding knowledge base on atypical vascular complications in surgical settings.

## Introduction

Postoperative bleeding, though infrequent, can have profound consequences on patient outcomes, necessitating a comprehensive exploration beyond routine considerations. Angiodysplasias, also known as vascular ectasias or angioectasias, are tortuous, dilated abnormal blood vessels of the mucosa and submucosa and are commonly found in the large bowel with a rare occurrence in the small bowel. They can be present in 8-23% of mid-lower gastrointestinal (GI) bleeding cases [1]. Large bowel angiodysplasias are often associated with renal failure and aortic stenosis, with a possible correlation with von Willebrand disease [2]. A rare contributor to bleeding complications other than surgical techniques, coagulation disorders, and vessel injuries, its clinical presentation, evaluation, and management options are discussed in this article.

## Case presentation

**Figure 1:**
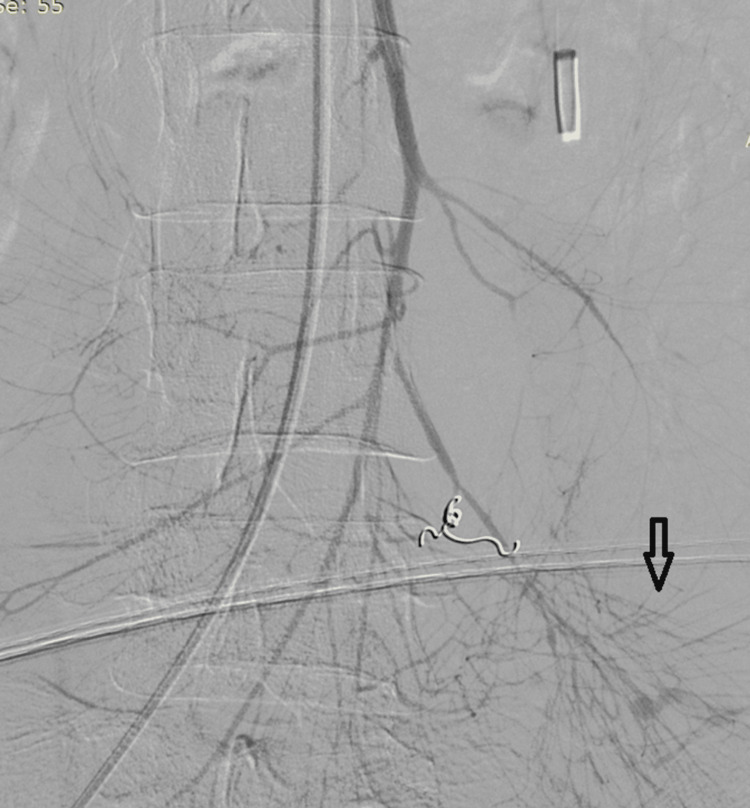
Abdominal angiogram showing a tortuous tubular lesion with contrast pooling suggestive of angiodysplasia (arrow).

**Figure 2:**
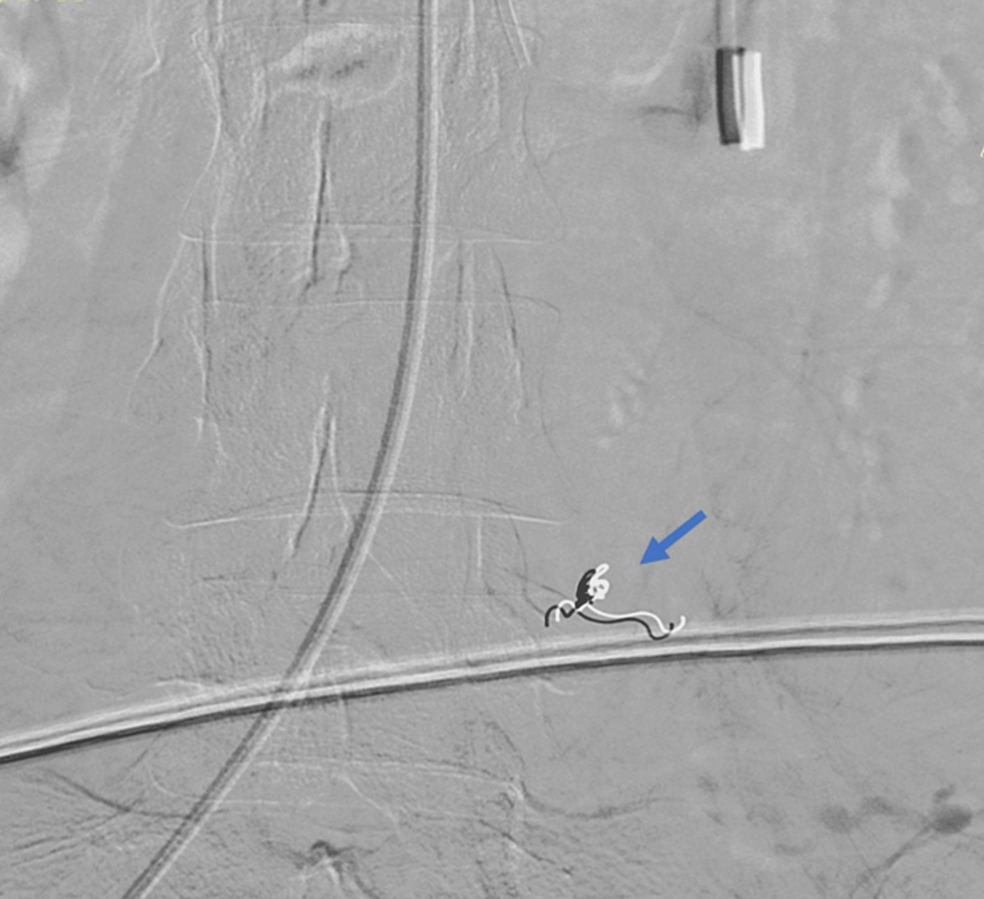
After angioembolization of the lesion (arrow shows the coil).

A female in her 20s was admitted as a case of jaundice after undergoing a laparoscopic cholecystectomy elsewhere. She was evaluated using magnetic resonance cholangiopancreatography and diagnosed as having type III benign biliary stricture (Bismuth-Corlette classification) which was managed by an open hepaticojejunostomy. The intraoperative and immediate postoperative periods were uneventful. On postoperative day four, the patient complained of hematemesis and melena. On examination, the nasogastric tube had an altered acid-hematin-like content and a sanguineous discharge from the drain. There was a serial drop in hematocrit from 34.7% to 26.1% to 20.3% over 36 hours, and the patient became hemodynamically unstable, requiring vasopressor support and blood transfusions. An urgent upper GI endoscopy was suggestive of probable bleeding beyond the D2-D3 junction. An abdominal angiogram revealed a normal portal vein and a tortuous tubular lesion with contrast pooling arising from the first jejunal arterial branch, suggestive of angiodysplasia of the Roux limb in the proximal limb (Figure 1). The patient was managed by CT-guided selective coil embolization of the affected feeding branch (Figure 2).

Post-procedure, the patient improved significantly and went off vasopressor support on day one of the procedure. The patient was discharged in fair general condition.

## Discussion

Postoperative hemorrhage can have dreaded complications ranging from requiring blood transfusion to requiring re-exploration to mortality; hence, it requires a quick, focused evaluation. Other than the common causes such as pseudoaneurysm and bleeding from the anastomotic site, angiodysplasias are a rare cause. Angiodysplasia is the most common vascular abnormality of the GI tract, with 77% located in the cecum and ascending colon, 15% in the jejunum and ileum, and 1-2% in the upper GI lesions. They are incidentally detected during colonoscopy in 0.8% of patients older than 50 years. They are responsible for 30-40% of cases of GI bleeding of obscure origin and 6% of cases of lower GI bleeding, but angiodysplasia presenting as postoperative bleeding is seldom reported [3].

Clinically, they usually present as low-grade bleeding in the form of hematochezia, melena, and massive bleeding in 15% of the patients; however, they can have rare presentations such as postoperative bleeding, as demonstrated in our case [4]. Iron deficiency anemia and stool positivity for occult blood can be the only presentations in 10-15% of patients. Bleeding is often recurrent and stops spontaneously in more than 90% of patients but can be fatal, requiring extreme methods of management, including embolization [4] and surgery, as shown in the index case.

The evaluation usually includes a colonoscopy with or without an upper GI endoscopy. Selective mesenteric angiography, which can detect bleeding as low as 0.5 mL/minute with a sensitivity of 58-86%, is used, especially in patients with massive GI bleeding, and colonoscopy is difficult [4]. The most frequent and earliest sign on angiography is a dilated, densely opacified, slow-emptying vein within the intestinal wall and a demonstration of extravasation of contrast dye in the bowel lumen if bleeding actively, as seen in the index case. Radionuclide scans using technetium-99m (99m Tc)-labeled red blood cells or 99m Tc sulfur colloid can detect bleeding as low as 0.1 mL/minute but are rarely used because of their low specificity [4]. Helical CT, wireless video capsule endoscopy, deep small bowel enteroscopy, intraoperative enteroscopy, and double balloon endoscopy are other options that can be used.

Treatment includes initial resuscitation, transfusion of blood products, and correction of coagulopathies. Endoscopic obliteration techniques such as monopolar electrocautery, heater probe, sclerotherapy, band ligation, and argon and neodymium: yttrium-aluminum-garnet lasers are commonly used for gastric and duodenal lesions, but the risk of rebleeding is high [5]. More commonly, super-selective embolization of visceral arterial branches using an injection of micro-coils, polyvinyl alcohol particles, gel form, or a selective vasopressin infusion achieves immediate cessation of bleeding in 97% of the cases, and the same was used in the index case. There is a 3% chance of post-embolization ischemia and 9% associated mortality in high-risk patients [5]. Baba et al. demonstrated the use of adult variable stiffness colonoscopy for post-hepaticojejunostomy jejunal afferent limb angiodysplasia inaccessible with conventional gastroduodenoscopy [6]. The treatment might require multiple modalities such as selective arterial embolization, simultaneous laparoscopic resection, and anastomosis of the bowel loop with angiodysplasia [7]. Medical treatment with somatostatin analogs, hormonal therapy, and thalidomide has been tried with limited success; hence, it was not tried in our case, where the patient was hemodynamically unstable. Surgical resection is the definitive treatment for small or large bowel angiodysplasia lesions that are not accessible to endoscopy or refractory to medical treatment.

## Conclusions

Angiodysplasias are a rare cause of GI bleeding and should be considered in the differential diagnosis of acute postoperative bleeding and patients being evaluated for obscure GI bleeding and chronic anemia, especially when conventional diagnostic modalities fail to provide a definitive answer. The successful outcome in this case, achieved through a multidisciplinary approach involving endoscopic intervention, underscores the importance of collaboration between gastroenterologists, radiologists, and surgeons.
